# Swimming during COVID-19: Operational recommendations and considerations for South African swimming venues

**DOI:** 10.17159/2078-516X/2020/v32i1a8993

**Published:** 2020-01-01

**Authors:** L Hill, P T Nikolaidis, B Knechtle

**Affiliations:** 1 Department of Exercise Science and Sports Medicine, University of Cape Town, Cape Town, South Africa; 2 Division of Gastroenterology and Nutrition, Department of Paediatrics, McMaster University, Hamilton, Canada; 3 School of Health and Caring Sciences, University of West Attica, Athens, Greece; 4 Institute of Primary Care, University of Zurich, Zurich, Switzerland; 5 Medbase St. Gallen Am Vadianplatz, St. Gallen, Switzerland

**Keywords:** swim, pandemic, operations, guidelines, safety

## Abstract

Swimming is one of the most popular recreational activities in South Africa. Since the emergence of the Coronavirus disease 2019 (COVID-19), South Africa imposed one of the strictest lockdown measures to contain and control the spread of the virus. These measures included the closure of gyms, fitness centres and swimming pools across the country. However, as the restrictions begin to ease, it is important to consider how swimming facilities can reopen whilst simultaneously ensuring appropriate measures are in place to reduce COVID-19 infections. Outlined are recommendations and considerations for swimming facilities in South Africa. Currently there is no evidence to suggest that COVID-19 transmission to humans is possible through water, making swimming one of the safer options for physical activity indoors. However, participation is still not without risk and compliance with government mandates and public health officials take precedent over the recommendations outlined in this article.

The emergence of the novel Coronavirus disease 2019 (COVID-19) has resulted in restrictive measures, such as generalised lockdown and mandatory quarantine across the world.^[1]^ In response, the government of South Africa imposed one of the strictest lockdown measures in order to contain and control the spread of the virus.^[2,3]^ Fitness centres, gyms, swimming pools other exercise venues were forced to close as part of the lockdown. ^[2,4]^ Infectious disease and public health specialists noted that all exercise venues were regarded as extremely high risk for the spreading of the virus. ^[5]^However, as lockdown and several other restrictions began to ease, it is important understand the specific public health issues and preventative measures required to operate and manage swimming facilities.^[6]^ It is still unclear whether possible contamination and transmission of COVID-19 can occur in swimming pools due to limited knowledge and the unpredictable behaviour of the virus.^[6]^ Swimming is one of the most popular recreational activities in South Africa^[7]^ and access to swimming pools, both indoors and outdoors, offers an extraordinary opportunity for the prevention of sedentary and lifestyle-related diseases that may have been induced by the extended lockdown. ^[5,6]^ As such, access to safe swimming spaces has an important role to play in facilitating various activities, including adapted physical activity for people of all ages, abilities and socioeconomic backgrounds. ^[8]^

The safe reopening of swimming facilities during lockdown and their appropriate management requires the implementation of policies and guidelines to ensure the well-being of the public.^[6]^ The current understanding of COVID-19 is continually advancing as research around its prevention and treatment proceeds.^[9]^ However, based on what is already known and working within the current public health guidelines, it is possible to define a framework of risks and outline potential preventive measures for sport. It is important to note that while COVID-19 is exceptionally contagious in most enclosed environments, this might not be the case in swimming pools. ^[10]^ According to epidemiological data presently available, there have been no reported cases of COVID-19 from swimming pools or through the use of water. ^[11]^

As swimming is such a popular sport, prevention of COVID-19 transmission in swimming pools must be examined. Although these environments may present a lesser risk of infection that others, caution is still necessary regarding the operation within public health recommendations and guidelines.^[12]^ This includes respecting current regulations and recognising that the understanding of the virus may change rapidly. Within this context, the government of South Africa already has specific requirements for maintaining swimming facilities regarding hygiene, sanitation, disinfection and good practice. ^[13]^ Recently, the South African Sports Medicine Association (SASMA) released a position statement for the resumption of sport within the country.^[3]^ The statement outlines five guidelines regarded as a minimum to be incorporated into any post-COVID-19 return-to-sport strategy. These include 1) education, 2) preparing the environment, 3) risk stratification of the specific sport, 4) risk stratification for the participants, and 5) the practical implementation of mitigating measures of different sports.^[3]^ Further, Swimming South Africa has also drafted COVID-19 compliance directives ^[14]^ which outline the guidelines and recommendations for the return to competitive swimming, training and learning to swim.

With these in mind, the potential safety considerations for swimming facilities are as follows:

1. Constant monitoring of the epidemiological status locally and countrywide.
2. Defining specific risk assessment based on general guidelines for the prevention of COVID-19. This includes the facility and building structure, the swimming pool area, as well as the changing rooms and service areas. This is the specific attention paid to the maximum occupancy for minimising transmission and hygiene protocols.
3. Acquisition and availability of personal protective equipment for staff, users and guests. This includes adherence to the Department of Health guidelines ^[12]^, including physical distancing, mask mandates and access to soap and hand sanitisers. For persons at the facility who are suspected to have COVID-19, a dedicated isolation room must be made available to allow for further assessment.
4. Preparation of a dedicated COVID-19 prevention plan which can be used on a daily basis as well as under extraordinary circumstances, such as the management of emergencies.
5. Individual facilities should create specific rules and guidelines in accordance with public health recommendations for the prevention of COVID-19 transmission. These should be readily available and made visible to users in order to reinforce specific hygiene rules and interpersonal distancing measures. Facilities should consider internal penalties for those who do not adhere to the specific rules or regulations. Further, facilities should implement mandatory temperature screening and the keeping of visitor logs which include contact details (for contact tracing purposes in the event of potential exposure).
6. Facilities should continually develop and appraise their preventative measures through dedicated surveillance and monitoring, in conjunction with staff training.
7. Due to the communicable nature of COVID-19, it is crucial that facilities determine appropriate measures for first aid and emergency interventions.

Key operational measures to ensure the safe function of swimming facilities should include those specifically outlined by the Department of Health ^[12]^ and other public health specialists. These measures (see below) are subject to change and largely dependent on the specific facility in question.

1. The organisation of effective access control to the facility, swimming pool area and change rooms for both staff, users, and visitors. Although already in place at most facilities, there should effective screening of COVID-19 symptoms prior to arrival at the venue and explicit prohibition of those who are likely to have COVID-19 symptoms, are awaiting test results for the virus or have recently come into contact with anyone who has tested positive for the virus.
2. This can be done through a self-assessment toolkit. ^[12]^
  - Common symptoms of COVID-19 may include but are not limited to:
    - Fever
    - Chills
    - New or worsening cough
    - Shortness of breath
    - Sore throat
    - Difficulty in swallowing
    - Decreased or loss of taste or smell
    - Unusual or long-lasting headache
    - Digestive issues such as nausea/vomiting, diarrhoea, stomach pain
    - Muscle aches that are unusual or long-lasting
    - Extreme tiredness
    - Sluggishness or lack of appetite
    - Coordination and balance issues
  - Considerations if a person falls into specific at-risk groups:
    - Getting treatment that compromises (weakens) the immune system
    - Having a condition that compromises (weakens) the immune system
    - Having a chronic (long-lasting) health condition
    - Regularly going to a hospital or healthcare facility for treatment
  - Being in close physical contact with someone who currently has COVID-19 (closer than one metre away from the infected person or in contact for more than 15 min, or medium risk >one metre or less than 15 min contact) ^[15]^ in the previous 14 days. Close physical contact with someone who currently has COVID-19 means any of the following:
    - being less than two metres away in the same room, workspace, or area
    - living in the same home
    - being in the same classroom
3. Within the exercise facility and in the pool, there must be encouragement for interpersonal distancing. This can largely be achieved through scheduled reservations, work shifts, one-way routes, or other means to prevent crowding within shared spaces. In the pool, it is important to limit a single person to a single lane. If not currently present, lane lines should be implemented with sufficient distance between them to maintain interpersonal separation.
4. Facilities should strive to assure mandated water quality levels through appropriate disinfection and hygiene requirements. Procedures should outline clear minimal requirements, including optimal residual disinfection activity and pH levels. Water management should be addressed through local and country-specific by-laws. Facilities should have in place effective and up-to-date water safety plans.
5. As COVID-19 is currently known to be transmitted through the air, indoor facilities should ensure adequate air quality and microclimates aiming for maximum safety. It is not sufficient to only adhere to the minimum required levels. Facilities should consider the best possible methods for achieving air exchange, internal circulation, and ventilation.
6. As recommended by public health officials, facilities should establish routine and appropriate protocols to ensure the sanitation of common areas, changing rooms and the pool area. Sanitation should be done on a frequent basis with greater attention paid to high touch areas such as door handles, handrails, and communal waiting areas. Sanitation should be conducted within the already available guidelines. Disinfection and cleaning protocols must be clearly defined and routinely conducted with periodic inspection to ensure correct execution and effectiveness.
7. Facility staff, users, and visitors must acknowledge the assumption of risk when utilising the swimming pool. Although the recommendations above may assist in the mitigation of transmission of COVID-19, it is essential to understand that no activity is risk-free. Therefore, a consent or waiver document should be in place prior to use of the swimming facility.

## The way forward

Although the above recommendations may assist with the mitigation of risk associated with a highly communicable infection like COVID-19, these recommendations should be considered as an addition to compliance to government and public health mandates. Appropriate adherence to the recommendations and bylaws should support efforts to reduce possible viral outbreaks, whilst simultaneously giving the public access to physical activity venues. ^[5,6]^ Whilst caution is still encouraged, the Italian Istituto Superiore di Sanità (National Health Institute) recently stated that there is no evidence that COVID-19 can be spread to humans through the use of swimming pools or whirlpools ^[6]^, further supported by the Centres for Disease Control and Prevention (CDC). ^[11]^ If disinfection with chlorine is done correctly, in conjunction with adequate maintenance, the Severe Acute Respiratory Syndrome coronavirus 2 (SARS-CoV-2) can be inactivated, reducing its infectivity and transmission. ^[10]^ Previously, SARS-CoV-1 was completely inactivated in wastewaters by chlorine (10 mg/L for 10 minutes with a concentration of free residual chlorine of 0.5 mg/L or with chlorine dioxide 40 mg/L for 30 minutes with a residual free chlorine concentration of 2.19 mg/L).^[10]^ Therefore, swimming may be one of the safer exercise activities the public can undertake as long as strict protocols are observed.

Nevertheless, independently from whether COVID-19 is transmissible or not in water, the recommendations should follow the general guidelines outlined by National and International Public Health agencies. A swimming pool is not only the water, but also a venue where people aggregate and are in close contact with each other both before and after swimming. Moreover, it is important to note that other risks are involved when travelling to facilities i.e. public transportation, which may also increase risk of COVID-19 exposure. It is necessary therefore to highlight the guidelines for swimming and pool venues should be done should be done within the context of the general guidelines of resumption of sport in South Africa.^[3]^

## Conclusion

Finally, the recommendations and considerations outlined within this commentary should assist owners and operators with reopening various facilities around the country. It is suggested that facilities employ a dedicated Compliance Officer whose sole task is the monitoring, evaluating, and organising the various preventative measures and their implementation.

## References

[1] Lancet COVID-19: too little, too late? Lancet 2020 395 10226 755 10.1016/S0140=6736(20)30522-5 32145772 PMC7135007

[2] Dyer O Covid-19: Africa records over 10 000 cases as lockdowns take hold BMJ 2020 369 m1439 10.1136/bmj.m1439 32269023

[3] Ramagole D Janse van Rensburg DC Pillay L Implications of COVID-19 for resumption of sport in South Africa: A South African Sports Medicine Association (SASMA) position statement SA J Sports Med 2020 32 1 1 6 10.17159/2078-516x/2020/v32i1a8484 PMC9924566 36818966

[4] Carmody S Murray A Borodina M When can professional sport recommence safely during the COVID-19 pandemic? Risk assessment and factors to consider Br J Sports Med 2020 54 16 946 948 10.1136/bjsports-2020-102539 32381501 PMC7418613

[5] Ding D Del Pozo Cruz B Green MA Is the COVID-19 lockdown nudging people to be more active: a big data analysis Br J Sports Med 2020 54 20 2019 2020 10.1136/bjsports-2020-102575 32605932

[6] Romano Spica V Gallè F Baldelli G Swimming Pool safety and prevention at the time of Covid-19: a consensus document from GSMS-SItI Ann Ig 2020 32 5 439 448 10.7416/ai.2020.2368 32578839

[7] Hill L Grand’Maison V Swimming, South Africa and the Olympics: A history of women’s participation In: Olympika: the international journal of Olympic studies 2017 26 36 52 Available: http:library.oluympic.org

[8] Chase NL Sui X Blair SN Comparison of the Health Aspects of Swimming With Other Types of Physical Activity and Sedentary Lifestyle Habits Int J Aquatic Res Educ 2008 2 2 Article 7 10.25035/ijare.02.02.07

[9] Yang Y Xiao Z Ye K SARS-CoV-2: characteristics and current advances in research Virol J 2020 17 117 10.1186/s12985-020-01369-z 32727485 PMC7387805

[10] La Rosa G Bonadonna L Lucentini L Coronavirus in water environments: Occurrence, persistence and concentration methods - A scoping review Water Res 2020 179 115899 10.1016/j.watres.2020.115899 32361598 PMC7187830

[11] Centers for Disease Control and Prevention Considerations for Public Pools, Hot Tubs, and Water Playgrounds during COVID-19 2020 CDC 24/7

[12] South Africa. Department of Health Corona Virus(COVID-19) Pandemic 2020 [cited 2020 Oct 7]. Available from: http://www.health.gov.za/index.php/outbreaks/145-corona-virus-outbreak/465-corona-virus-outbreak

[13] South Africa Occupational Health and Safety Act Government Gazette 1993,337 no.14918 [cited 2020 Oct 7]. Available from: https://www.gov.za/documents/occupational-health-and-safety-act

[14] Swimming South Africa SSA Directive: Ensuring a safe aquatic environment and protecting the health of members 2020; SSA COVID-19 Directive 01 9 June 2020 [cited 2020 Oct 9]. Available from: http://swimsa.org/about/correspondence/general-news/directive/view

[15] Mann RH Clift BC Boykoff J Athletes as community; athletes in community: Covid-19, sporting mega-events and athlete health protection Br J Sports Med 2020 54 18 1071 1072 10.1136/bjsports-2020-102433 32303522 PMC7497562

